# Supplementary material for: Development and External Validation of a PET Radiomic Model for Prognostication of Head and Neck Cancer
Source: Cancers (Basel). 2023 May 9;15(10):2681. doi: 10.3390/cancers15102681 (PMC10216021; doi:10.3390/cancers15102681)
Supplement: Supplementary file 1 [file cancers-15-02681-s001.zip › cancers-2221976-supplementary.pdf]

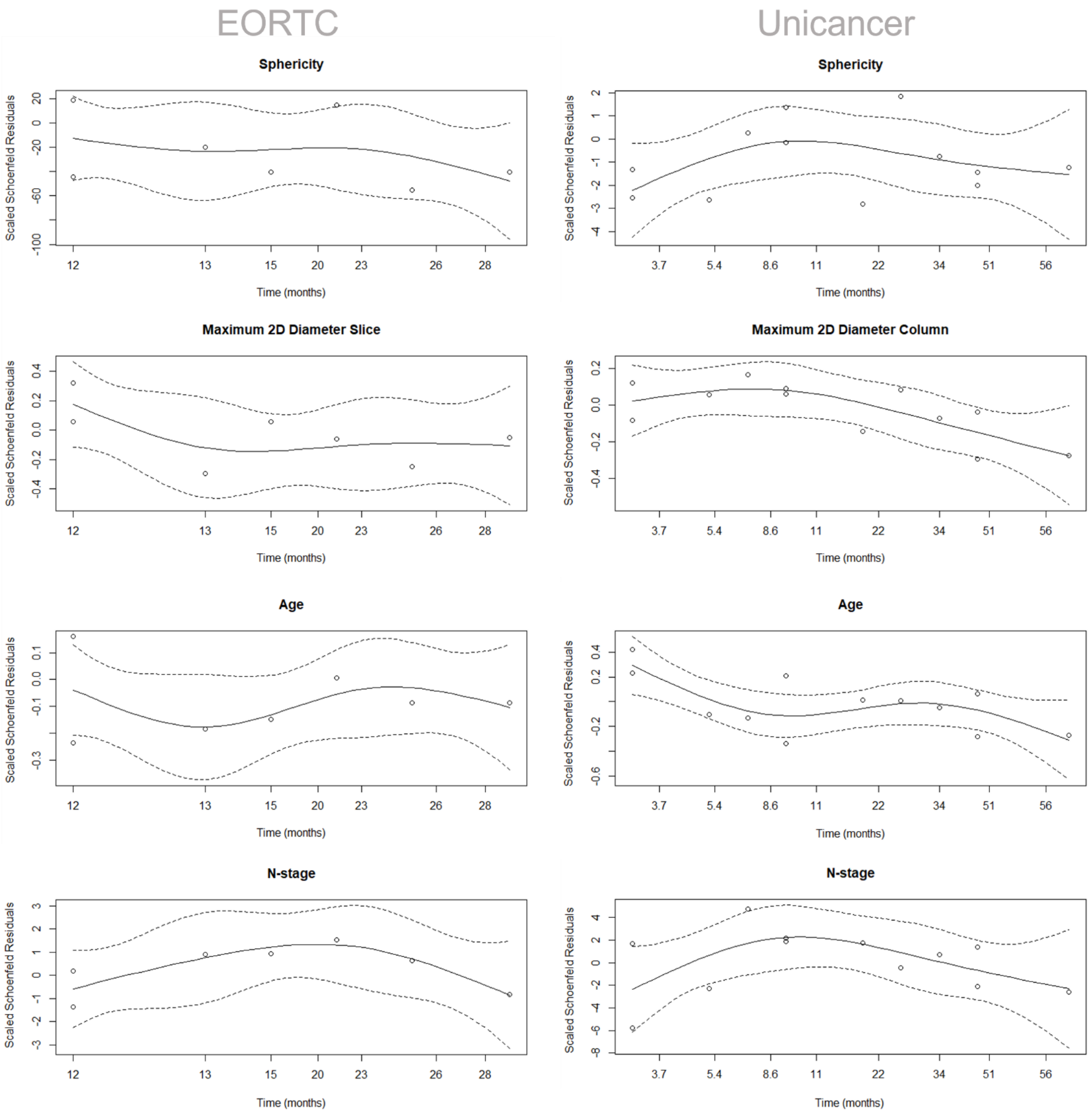

Supplementary Figure S1. Proportional hazard assumption test for overall survival by plotting the Schoenfeld residuals against time for both the EORTC and Unicancer cohort. The x-axis represents the survival time in months; the y-axis represents the scaled Schoenfield residuals for sphericity, maximum 2D diameter slice/column, age, and N-stage.
